# From Illness to Resilience: Mediating Factors of Quality of Life in Patients with Congenital Heart Disease

**DOI:** 10.31083/j.rcm2408224

**Published:** 2023-08-01

**Authors:** Fernanda Moedas, Filipa Nunes, Paula Brito, Ana Bessa, Sara Espírito Santo, Sara Soares, Marisa Pereira, Victor Viana, Bruno Peixoto, Joana O Miranda, José Carlos Areias, Maria Emília G. Areias

**Affiliations:** ^1^Department of Social and Behavioral Sciences of the University Institute of Health Sciences-CESPU, 4585-116 Gandra, Portugal; ^2^Department of Pediatric Cardiology, University Hospital Center S. João, 4200-319 Porto, Portugal; ^3^Department of Pediatrics, University Hospital Center S. João, 4200-319 Porto, Portugal; ^4^Faculty of Nutrition and Food Sciences, University of Porto, 4150-180 Porto, Portugal; ^5^CINTESIS, Faculty of Medicine, University of Porto, 4050-526 Porto, Portugal; ^6^UnIC@RISE, Faculty of Medicine, University of Porto, 4050-526 Porto, Portugal

**Keywords:** congenital heart disease, severity, quality of life, number of surgical interventions, mediation models

## Abstract

**Background:**

Congenital heart disease (CHD) is a leading cause of childhood morbidity, with an estimated prevalence of 0.8–1%. However, advances in diagnosis and treatment now allow 90% of childhood CHD patients to survive to adulthood, leading to increased interest in their quality of life (QoL). In this study, we examine the impact of clinical and psychosocial variables, including the number of surgical interventions (NSI), age at surgery, school achievement, and social support, as mediating factors of QoL in CHD patients.

**Methods:**

The study included 233 CHD patients (132 males) with an average age of 15.2 ± 2.07 years, including 80 with cyanotic CHD and 153 with acyanotic CHD. The severity of illness ranged from mild to severe, with 30 patients having a severe illness, 119 having a moderate illness, and 84 having a mild illness. One-hundred-sixty-three patients underwent surgery. Clinical data on diagnosis, the severity of CHD, the type of CHD, and surgical interventions were collected from patient records, and a semi-structured interview was conducted to explore the relationship between CHD diagnosis and various aspects of life. QoL was assessed using the Abbreviated World Health Organization Quality of Life questionnaire (WHOQOL-Bref) questionnaire.

**Results:**

Ten mediation models were analyzed, each with three hypotheses (paths). In all models the first hypothesis was supported. Analyses of the second and third hypotheses revealed three feasible models of mediation through the effect of NSI on QoL in CHD patients.

**Conclusions:**

Our findings indicate that patients with more severe and cyanotic CHD generally require more surgical interventions, which may increase the risk of negative outcomes and affect patients’ perception of QoL. These results have important implications for healthcare providers and psychologists who work with childhood CHD patients.

## 1. Introduction 

This study is part of a broad research line dedicated to the study of congenital heart disease (CHD) in adolescents and young adults, which aims to understand the impact of the disease on quality of life (QoL), psychosocial adjustment, neurocognitive performance, as well as on associated psychiatric morbidity.

CHD is currently the leading cause of childhood morbidity. Estimates of its current prevalence are between 1 case in 100 births (1%) [1, 2, 3] and 5 to 8 cases in 1000 births (0.8%) [4]. In the 1950s, only 20% of children born with moderate or severe CHD (such as tetralogy of Fallot, transposition of the great arteries, and hypoplastic left heart syndrome) survived the first year of life [4]. Over the last decades, with progress in diagnosis and surgical conditions, 90% of patients with CHD survive to adulthood. That represents a new challenge, as blood flow and hypoxia during critical phases of fetal brain development may have irreversible consequences, leading to life-long cognitive impairment and generating interest in the study of their psychosocial adjustment, psychiatric morbidity, QoL, and their Neurocognitive performance [2, 5, 6, 7, 8, 9, 10, 11, 12, 13, 14, 15].

The diagnostic and therapeutic advances in CHD have contributed to decreased infant mortality and an increasing number of adolescents and adults with CHD. CHD is currently considered a chronic disease, so these patients face several difficulties in several domains. Survival does not always mean high QoL, with the need for hospitalization or interruption of pleasurable activities [3]. As a result, one of the key aspects for assessing healthcare impact outcomes in these patients is the study of variables associated with functional health, such as exercise capacity, or variables related to health indicators (such as a cardiopulmonary function) related to the patient’s QoL [16, 17, 18]. In addition, QoL is now widely used in exploratory studies on the efficacy of treatment methods from the patient’s point of view [19] to serve as the basis for guiding the decisions of professionals and patients at the level of the most appropriate healthcare. The definition of health by the World Health Organization (WHO) [20] implies that assessments of health status and their effects also include indicators of well-being, which can be achieved by evaluating health-related QoL.

The WHO defines QoL as the individual’s perception of their position in life in the context of their culture, the value system in which they live and their goals, expectations, standards, and concerns. QoL is a comprehensive concept that is affected by physical health, psychological state, personal beliefs, social relations, and its relationship with the salient characteristics of its environment [3, 20]. Studies have evaluated the relationship between health, illness, frequent hospitalizations, medical therapy, or health care with QoL in the patient’s physical, social, or psychological functioning [21]. The results in the physical domain of QoL turned out to be lower in adolescents with tetralogy of Fallot compared to healthy adolescents and in the domain of social relations when associated with executive dysfunctions associated with attention deficit hyperactivity disorder [22]. Some studies have found that children and adolescents with CHD show symptomatology of anxiety and/or depression related to the limitations imposed by the disease, frequent hospitalizations and, in some cases, the need for regular medication [3], as well as changes in body image in the postoperative period (which often leaves a large scar on the chest). As a result, these children become more introverted and isolated from others because they feel shame and guilt for their body image. One reason for poor QoL is the lack of social acceptance, especially in the school setting [3]. Wernovsky [23] have studied the school performance of these children and adolescents with CHD, which tends to be marked by various irregularities, such as learning difficulties, poor performance, behaviour problems, reduced socialization skills, low self-esteem and, in less frequent cases, delinquency, absenteeism, due to hospital admissions, surgeries and frequent treatments. One of the reasons for having a poor QoL is the lack of social acceptance, especially in the school environment [3]. Physical activity restrictions have an impact on the QoL of children with CHD, whether they are the same as those imposed by the disease condition, which reduces the opportunity to enjoy the benefits of physical activity for mental health [24], or by parents, who are often overprotective [3]. Patients often report problems such as shortness of breath, tiredness, chest pain, and dizziness during exercise [19].

Patients with CHD are regularly followed up at health services, where certain clinical variables (presence or absence of cyanosis, the severity of disease, surgical interventions, need for pharmacological therapy, presence of residual lesions) can help understand the situation and to ensure the best care. Among the variables above, the surgical interventions (their need, their quantity) may impact the perception of QoL, mainly in the physical domain [25]. Several studies have shown that newborns with CHD have a risk of neurodevelopmental changes before surgery, confirmed by neuroimaging [4, 26]. These changes are observed in the preoperative phase, suggesting the presence of cerebral anomalies in children with CHD [4]. Magnetic resonance imaging of the brain, performed before surgery, demonstrates a high incidence of preoperative brain injuries, such as corneal agenesis, holoprosencephaly, microcephaly, lissencephaly, Dandy-Walker malformation, and immature cortical mantle [5].

There has been much debate about the effect of cardiac surgery (with thoracic cavity opening) on neurocognitive performance in children and adolescents with CHD [13, 27, 28, 29].

In addition to the studies on the impact of surgical interventions on the health-related medical conditions of patients with CHD, some studies already focus on the QoL of these patients [30, 31]. The impact of the implantation of a prosthetic heart valve on the QoL has been studied since these patients are confronted with specific postoperative problems, such as the need for anticoagulants, the expected problems in future pregnancies (in the case of women), and the new operations provided by the prosthetic valve degeneration [30, 32].

As several of these consequences may arise from mild, moderate, or severe forms of CHD, it is crucial to plan adequate clinical resources and support to understand the underlying mechanisms that may explain different patterns of adaptation in patients, some fostering resilience and others increasing detrimental effects.

Therefore, in this study, we intended to test several mediation mechanisms between the severity of illness, the presence of cyanosis, and the QoL of patients. We hypothesized that some clinical (number of surgical interventions (NSI), age at first surgery) and psychosocial variables (school achievement, social support) might be mediators of the impact of illness in QoL in its different domains (physical, psychological, social relationships, environmental, and general).

Thus, this study examines the importance of selected clinical variables (NSI) in mediating the impact of CHD (namely severity and presence/absence of cyanosis) on the perception of QoL in patients.

## 2. Methods 

To pursue this aim, we started by considering the following research hypotheses: (1) NSI has a mediating mechanism of impact, influencing the effect of type of congenital heart disease (TCHD) and decreasing the perception of QoL in patients with the cyanotic disease; and (2) NSI has a mediating mechanism of impact, influencing the effect of severity of congenital heart disease (SCHD) and decreasing the perception of QoL in patients with more severe diseases.

### 2.1 Models 

Then, we operationalized these main hypotheses in possible models of mediation. For each possible model, we defined and tested three paths (a, b, c) according to mediation meaning: (a) variations in the levels of the independent variable significantly give rise to variations in the presumed mediator; (b) variations in the mediator significantly give rise to variations in the dependent variable; and (c) when paths a and b are controlled, the previously significant relationship between the independent and dependent variables is no longer significant.

We are interested in better understanding how the severity and/or presence of cyanosis in CHD affects the QoL of patients. Therefore, we used mediation analysis to investigate the perception of QoL in patients with CHD.

### 2.2 Hypotheses

**Fig. 1.**
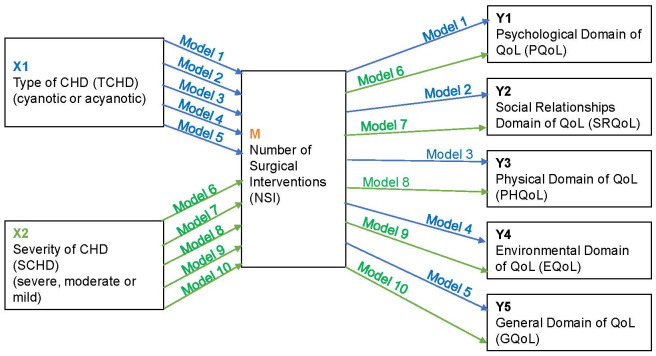
**Diagram of mediation models**. Blue: Models considering variable X1: Type of CHD (TCHD); Green: Models considering variable X2: Severity of CHD (SCHD). CHD, congenital heart disease; QoL, quality of life.

Fig. 1 presents a diagram with the ten mediation models that we tested for the criterion variable “QoL”.

For each model, we tested three hypotheses:

Hypothesis 1: TCHD, the presence of cyanotic or acyanotic disease, or SCHD (severe, moderate, or mild), and NSI are positively related.

Hypothesis 2: NSI is negatively related to a specific domain or general QoL in patients with CHD.

Hypothesis 3: TCHD or SCHD is sequentially positively associated with NSI and is also associated with a decreased perception of a specific domain or general QoL. Estimating the indirect effect of the mediator (NSI) using the bias-corrected bootstrap method produces a confidence interval that does not include zero.

Model 1: Predictive mediator effect of NSI on patients’ perception of the psychological domain in QoL (PQoL) according to TCHD.

Model 2: Predictive mediator effect of NSI on the perception of social relationships domain in QoL (SRQoL) of patients according to TCHD.

Model 3: Predictive mediator effect of NSI on patients’ perception of the physical domain in QoL (PHQoL) according to TCHD.

Model 4: Predictive mediator effect of NSI on patients’ perception of the environmental domain in QoL (EQoL) according to TCHD.

Model 5: Predictive mediator effect of NSI on patients’ perception of the general domain in QoL (GQoL) according to TCHD.

Model 6: Predictive mediator effect of NSI on the perception of PQoL of patients according to SCHD.

Model 7: Predictive mediator effect of NSI on the perception of SRQoL of patients according to SCHD.

Model 8: Predictive mediator effect of NSI on the perception of PHQoL of patients according to SCHD.

Model 9: Predictive mediator effect of NSI on the perception of EQoL of patients according to SCHD.

Model 10: Predictive mediator effect of NSI on the perception of GQoL of patients according to SCHD.

### 2.3 Participants

Participants were recruited consecutively at a tertiary university hospital’s outpatient pediatric cardiology clinic in northern Portugal. We included only patients with complete medical records, aged between 12 and 25 years, and the necessary basic educational level to understand and complete the written questionnaires. We excluded those patients with associated extracardiac malformations, mental or physical comorbidity, or chromosomal disorders that might have associated cognitive development problems. Of all patients invited, only nine refused to participate. Three-hundred-ninety-three patients participated in the study, but only 233 completed the protocol, considering neurocognitive variables and neonatal markers in fetal development. According to Table 1 we included only patients with complete medical records, aged between 12 and 25 years. Table 2 describes the distribution of participants according to the different clinical variables considered.

**Table 1.** **Sociodemographic characteristics of the participants**.

Sociodemographic characteristics		Patients N = 233
Sex	Male	132
	Female	101
Age (in years)	Range	12–21
	(M ± SD)	15.2 ± 2.07
Years completed at school		9.4 ± 2.03
Years completed at school by father		9.7 ± 4.05
Years completed at school by mother		10.3 ± 4.02
Completed education	2nd cycle	27
	3rd cycle	116
	Secondary level	84
	University degree	5
Retentions at school	Number of patients	69
	Years of retention (M ± SD)	0.5 ± 0.9
Marital Status	Single	233
	Married	0
	Divorced	0
	Living in marital union	0

N, No. of patients; M, mean; SD, standard deviation.

**Table 2.** **Distribution of participants on clinical variables**.

Clinical variables		Number of patients (N = 233)
Age when diagnosed	Neonatal period	138
	Until 1 year	49
	1–3 years	13
	3–6 years	12
	6–12 years	15
	12–18 years	6
Severity of CHD	Severe	30
	Moderate	119
	Mild	84
Type of CHD	Cyanotic	80
	Acyanotic	153
Residual lesions	Severe/moderate	27
	Mild	138
	Without	68
Intensive care	Yes	163
	No	70
Physical limitations	Physical limitations	85
	Satisfactory physical competence	148
Pharmacological therapy	Yes	57
	No	176
Surgical interventions	Yes	163
	No	70
Number of surgical interventions	0	70
	1	109
	2–4	50
	5–8	3
	10 or more	1
Age at first surgery	Neonatal period	68
	7 months until 1 year	28
	1–3 years	23
	3–6 years	18
	6–12 years	20
	12–18 years	6
	Without surgery	70

CHD, congenital heart disease.

### 2.4 Measures and Analysis 

Relevant clinical data were collected retrospectively using each patient’s clinical record, including diagnosis, severity, category of CHD and surgical interventions, pharmacological therapy, and presence of residual lesions. Personal and demographic data were collected using a semi-structured interview focused on the relationship between the diagnosis of CHD and the various aspects of life. We used the Portuguese translation of the self-report questionnaire of the WHO (the WHOQOL-BREF) to assess subjective quality of life. This questionnaire is adapted to the general Portuguese population [33]. This questionnaire includes 26 questions, and the answers are filled in options of a Likert scale type, ranging from 1 to 5, where higher scores reveal a higher QoL, except for questions 3, 4, and 26, which are formulated inversely, and the scale is also inverted. The WHOQOL-Bref is organized into four domains of QoL: Physical (questions 3, 4, 10, 15, 16, 17, and 18), Psychological (questions 5, 6, 7, 11, 19 and 26), Social Relationships (questions 20, 21, and 22), and Environment (questions 8, 9, 12, 13, 14, 23, 24, and 25). Besides those, there is also an overall indicator, the General QoL, which includes the first two questions of the questionnaire. For each domain, the average of the scores needs to be calculated, and finally, the results are transformed into a scale from 0 to 100.

Additional questionnaires and evaluations were used in this research and are detailed in another paper. A neuropsychological evaluation was carried out to evaluate the performance of different neurocognitive functions that the literature has shown may affect CHD patients [34, 35]. We used the NEO Five-Factor Inventory (NEO-FFI, reduced version), a self-report questionnaire that provides data to access personality traits in five domains (Neuroticism, Extroversion, Openness to Experience, Kindness, and Responsibility). We also used a standardized psychiatric interview (SADS-L) for the clinical diagnosis of psychopathological disorders, covering the patient’s lifetime up to the moment of the interview.

### 2.5 Design 

The study design is cross-sectional, with all the assessments being performed simultaneously. The patient’s medical history was collected retrospectively with the collaboration of the medical and administrative staff.

### 2.6 Statistical Analyses 

Statistical analyzes were performed using the IBM SPSS Statistics for Windows program, version 27 (IBM Corp., Armonk, NY, USA). For the characterization of the participants, we used descriptive statistics. Regarding the variable TCHD (cyanotic and acyanotic), to ensure that the groups would be equivalent in the main demographic variables, we compared parents’ schooling using Student’s *t*-test.

To test the mediation hypotheses, we used Hayes’s PROCESS version 3.5.3 (http://www.processmacro.org) [36] (model 4) for SPSS using 5000 bootstrap simulations to calculate the total direct and indirect effects of the variables. Unstandardized coefficients were used to test each model’s first and second hypotheses. The point estimate of the specific indirect effect through the mediator was performed as a test of the third hypothesis of each model.

## 3. Results 

A total of 233 patients were enrolled in the study, comprising 132 males and 101 females aged between 12 and 21 years (mean age = 15.2 ± 2.07), with a mean of 9.4 ± 2.03 years of schooling (Table 1). The average schooling of the father and mother was 9.7 ± 4.05 years and 10.3 ± 4.02 years, respectively. At the time of the interview, 27 patients had completed the 2nd cycle of elementary education, 116 had completed the 3rd cycle, 84 had completed secondary education, and 5 had higher education. Sixty-nine patients had experienced schooling retention, with an average retention of 0.5 years (+0.9). All 233 participants were single. Table 2 presents the description of the clinical variables. Among our participants, 138 were diagnosed with CHD in the neonatal period, 49 were diagnosed up to 1 year, 13 were diagnosed between 1 and 3 years of age, 12 between 3 and 6 years, 15 between 6 and 12 years, and 6 between 12 and 18 years. The diagnoses of CHD were classified as cyanotic (80 patients) or acyanotic (153 patients) based on cyanosis in the original cardiac malformation. The severity of CHD at the time of diagnosis was classified as severe (30 patients), moderate (119 patients), or mild (84 patients) according to the clinical processes. Considering the impact of the disease on patient performance, 85 patients had physical limitations, while 148 had no physical limitations. The frequency of different pathologies according to the main diagnosis is shown in Table 3, with the most frequent pathologies being Ventricular Septal Defect (48 patients), Tetralogy of Fallot (36 patients), Coarctation of the Aorta (23 patients), and Atrial Septal Defect (21 patients). Some patients had one of these diagnoses, while others presented comorbidity among different cardiac pathologies. A total of 163 patients required surgical intervention, while 70 had no surgical intervention. Among those who underwent surgical interventions, 109 had one intervention, 50 had 2 to 4, 3 had 5–8, and 1 had 10 or more surgeries. The age at the first surgery among the patients who underwent surgical interventions was, for 68 patients, the neonatal period up to 6 months of age. For 28 patients, the first intervention occurred between 7 months and 1 year of age. For 23 participants, it occurred between 1 year and 3 years, 18 patients between 3 and 6 years, 20 patients between 6 and 12 years, and 6 patients between 12 and 18 years. Of the total participants in our study, 57 required pharmacological therapy (Table 2).

**Table 3.** **Distribution of participants according to the main diagnosis of CHD**.

Main diagnosis	Number of patients (N = 233)
Ventricular septal defect	48
Atrial septal defect	21
Atrioventricular septal defect	6
Coarctation of the aorta	23
Pulmonary stenosis	19
Aortic stenosis	7
Dysplastic pulmonary valve	2
Bicuspid aortic valve	10
Mitral valve prolapse	4
Ductus arteriosus	1
Dilated coronary sinus	1
Dilated cardiomyopathy	3
Tetralogy of fallot	36
Transposition of the great arteries	31
Anomalous pulmonary venous drainage	4
Pulmonary atresia	5
Tricuspid atresia	3
Double outlet right ventricle	3
Truncus arteriosus	2
Univentricular heart	2
Hypoplastic left heart ventricle	1
Not specified	1

CHD, congenital heart disease.

Demographic characteristics of parents’ education were compared between cyanotic and acyanotic CHD patients. The Levene test was performed to assess the assumption of variance between the two groups for both father’s and mother’s schooling, and the results validated the hypothesis that variance was equal in both groups for the Student’s *t*-tests. The *t*-test results demonstrated that the groups were equivalent in terms of demographic variables, and no statistically significant difference was found between the groups (father’s education: t = –1.114, *p* = 0.267; mother’s education: t = 1.458, *p* = 0.147).

### Mediation Models 

We analyzed ten mediation models to determine the predictive mediator effect of NSI on QoL in patients with CHD. The first hypothesis was supported for all ten mediation models, with TCHD and SCHD showing a statistically significant relationship with NSI. The second hypothesis, which states that NSI is negatively related to QoL, was supported in four models: Model 1 (TCHD- > NSI- > Psychological Domain), Model 2 (TCHD- > NSI- > Social Relationships Domain), Model 6 (SCHD- > NSI- > Psychological Domain), and Model 7 (SCHD- > NSI- > Social Relationships Domain). The third hypothesis, stating that TCHD or SCHD is sequentially positively associated with NSI and is also associated with a decreased perception of QoL, was supported in Models 1, 2, and 6. Out of the ten models, we found three feasible mediation models through the effect of NSI on QoL in CHD patients: Model 1, Model 2, and Model 6, where each of the three hypotheses was supported.

**Model 1**: Mediating effect of NSI between TCHD and the perception of PQoL of patients.

**Model 2**: Mediating effect of NSI between TCHD and the perception of SRQoL of patients

Table 4 and Appendix Fig. 2 show that independent variable X [TCHD (cyanotic or acyanotic)] has a statistically significant relationship with the mediator variable (M): NSI (B = –0.8520, size of effect (SE) = 0.1856, t = –4.5902, *p* <0.001), supporting hypothesis 1. The B value is negative because of the characteristics of variable X (discreet), but the meaning of the statistical relationship is positive: the presence of cyanosis is positively associated with statistical significance to the increase in NSI. NSI is negatively correlated in a statistically significant way with PQoL (B = –2.1444, SE = 0.8018, t = –2.6746, *p* < 0.01) (an increase in NSI corresponds to a decrease in PQoL), supporting hypothesis 2.

**Table 4.** **Total, direct and indirect effects (Model 1)**.

Predictive mediator effect of the number of surgical interventions (NSI) on the perception of Psychological Domain in QoL (PQoL) of patients according to Type of Congenital Heart Disease (TCHD)							
		Coeff	SE	t	*p*	LLCI	ULCI
Path a	TCHD (X)	–0.8520	0.1856	–4.5902^***^	0.0000	–1.2182	–0.4858
Path b	NSI (M)	–2.1444	0.8018	–2.6746^**^	0.0082	–3.7264	–0.5625
Path c	Total effect of X on Y	1.3570	2.0468	0.6630	0.5082	–2.6814	5.3954
Path c’	Direct effect of M on Y	–0.4701	2.1260	–0.2211	0.8252	–4.6648	3.7246
Indirect effect of X on Y ab 95% bootstrap confidence interval		Effect	BootSE				BootLLCI
NSI		1.8271	0.9954			0.3121	4.2045

SE, size of effect; LLCI, lower limit of confidence interval; ULCI, upper limit of confidence interval; QoL, quality of life; Boot, resampling simulation. The number of sample simulations for bias correction of confidence intervals: Level of confidence for all confidence intervals: 95. ^**^p < 0.01; ^***^p < 0.001.

**Fig. 2.**
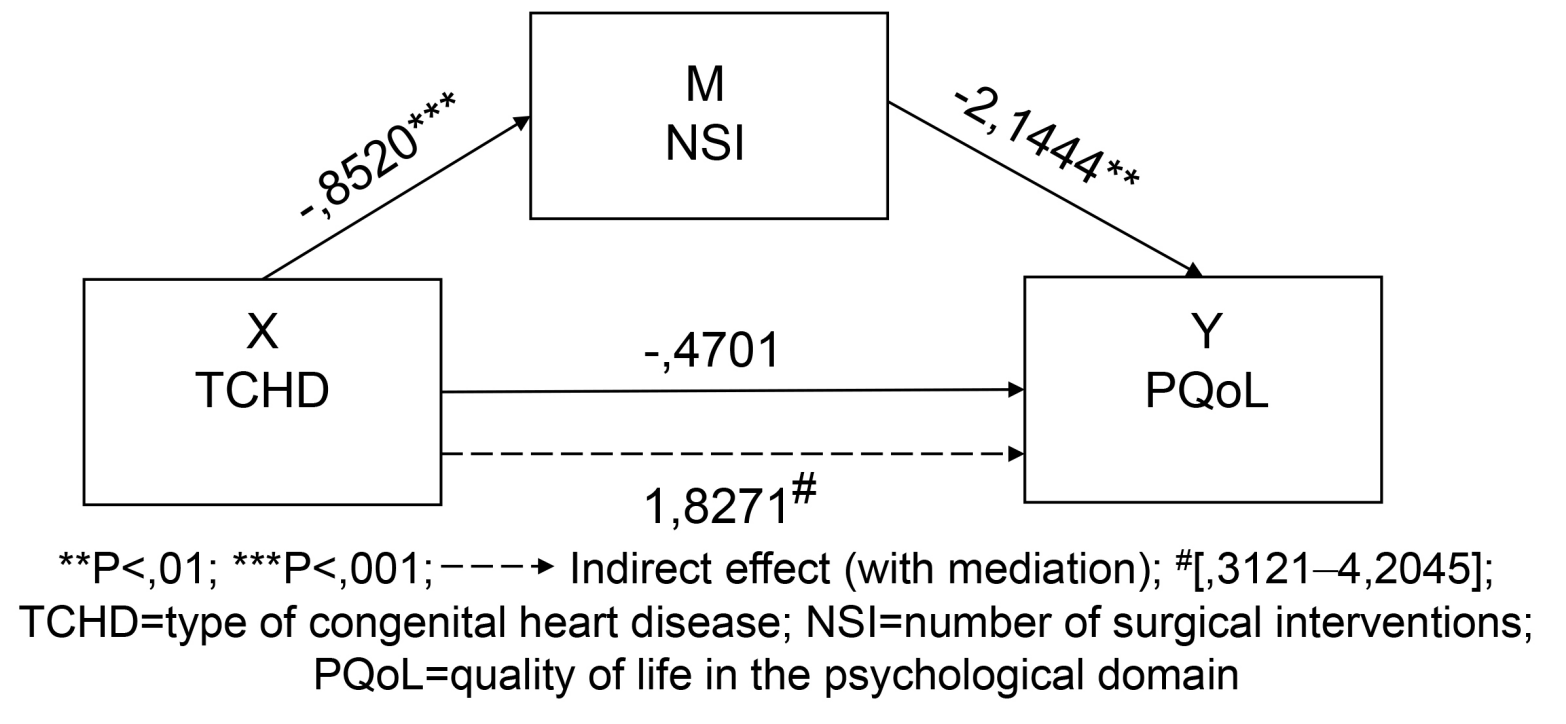
**Model 1: unstandardized path coefficients for mediation**.

The direct effect between variables X: TCHD and Y: PQoL shows a positive association (B = –0.4701, SE = 2.1260, t = –0.2211, *p* > 0.05), although this relationship is not statistically significant (the B value is negative because of the characteristics of variable X: discreet, but the meaning of the statistical relation is positive). As for the point estimate of the specific indirect effect through the mediator variable M (NSI) (X- > M- > Y), we have an estimated indirect effect value = 1.8271, which points in the direction of a decrease in PQoL (values show an increase, but the statistical significance of this relation corresponds to a decrease, due to the characteristics of variable X: discreet, supporting hypothesis 3. The bias-adjusted confidence interval of the product of this relationship between variables of 95%, calculated using the resampling simulation method between 3121 and 42,045, does not include zero, thus rejecting the null hypothesis and providing evidence of a significant mediator effect, as well as the relevance of Model 1 of mediation.

**Model 2**: Mediating effect of NSI between TCHD and the perception of SRQoL of patients.

Table 5 and Appendix Fig. 3 show that hypothesis 1 is corroborated (the presence of cyanosis is positively associated with statistical significance to the increase in NSI). NSI is negatively correlated in a statistically significant way with SRQoL (B = –2.0814, SE = 0.8179, t = –2.5448, *p* < 0.05) (an increase in NSI corresponds to a decrease in SRQoL), supporting hypothesis 2.

**Table 5.** **Total, direct and indirect effects (Model 2)**.

Predictive mediator effect of the number of surgical interventions (NSI) on the perception of Social Relationships Domain in QoL (SRQoL) of patients according to Type of Congenital Heart Disease (TCHD)							
		Coeff	SE	T	*p*	LLCI	ULCI
Path a	TCHD (X)	–0.8520	0.1856	–4.5902^***^	0.0000	–1.2182	–0.4858
Path b	NSI (M)	–2.0814	0.8179	${\mathrm{–2.5448}}^{*}$	0.0118	–3.6952	–0.4676
Path c	Total effect of X on Y	–1.5928	2.0842	0.7642	0.4457	–2.5194	5.7050
Path c’	Direct effect of M on Y	–0.1806	2.1687	–0.0833	0.9337	–4.4597	4.0985
Indirect effect of X on Y ab 95% bootstrap confidence interval		Effect	BootSE				BootLLCI
NSI		1.7734	0.9804			0.0139	0.2983

SE, size of effect; LLCI, lower limit of confidence interval; ULCI, upper limit of confidence interval; QoL, quality of life; Boot, resampling simulation. The number of sample simulations for bias correction of confidence intervals: Level of confidence for all confidence intervals: 95. ^*^p < 0.05; ^***^p < 0.001.

**Fig. 3.**
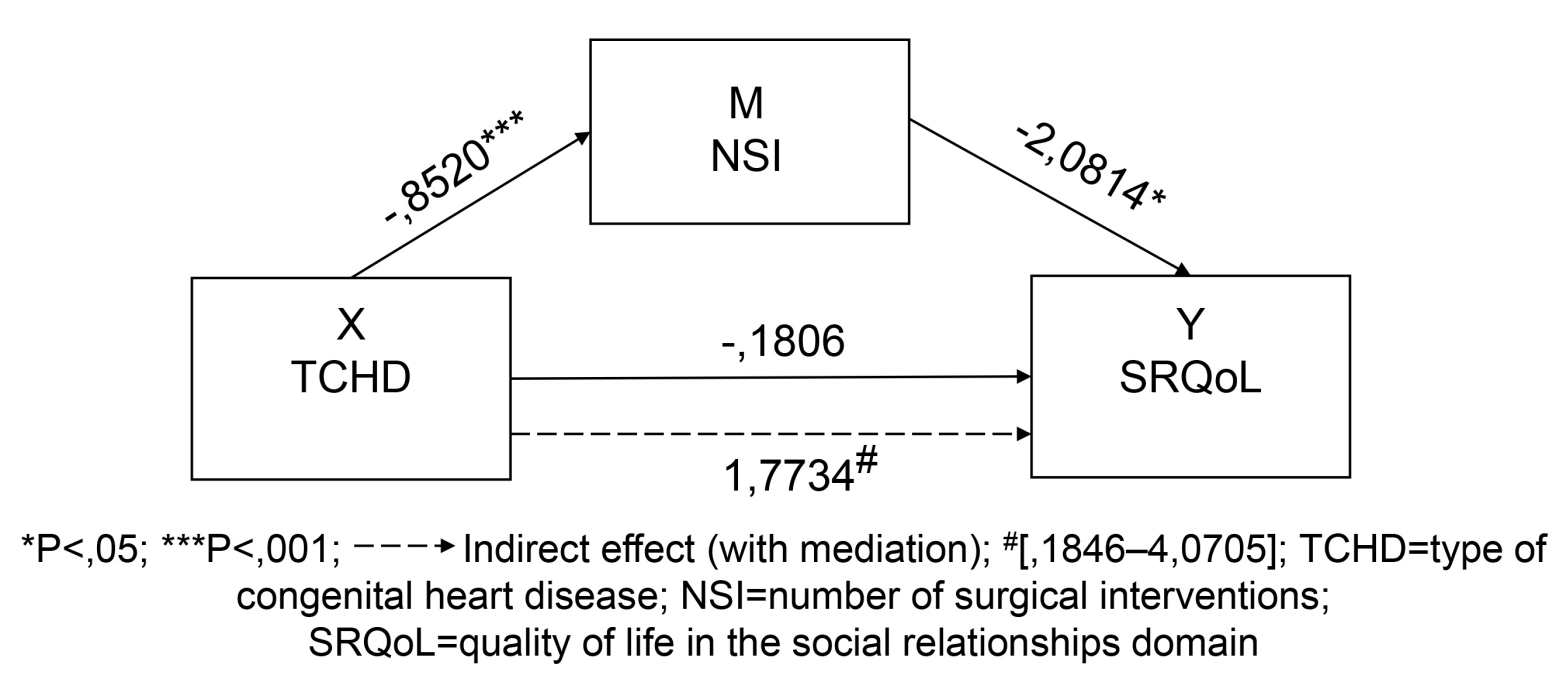
**Model 2: unstandardized path coefficients for mediation**.

The direct effect between the variables X: TCHD and Y: SRQoL shows a positive association (B = –0.1806, SE = 2.1687, t = –0.0833, *p* > 0.05), although this relationship is not statistically significant (the B value is negative because of the characteristics of variable X: discreet, but the meaning of the statistical relation is positive). As for the point estimate of the specific indirect effect through the mediator variable M (NSI) (X- > M- > Y), the estimated indirect effect = 1.7734 points towards a decrease in SRQoL: the numerical values reveal an increase, but the statistical significance of this relation corresponds to a decrease, due to the characteristics of the variable X: discreet, supporting the hypothesis 3. The bias-adjusted confidence interval of the product of this relation between variables of 95%, calculated according to the resampling simulation method between 1846 and 40,705, does not include zero, thus rejecting the null hypothesis and providing evidence of a significant mediator effect, as well as of the pertinence of Model 2 of mediation.

**Model 6**: Mediating effect of NSI between SCHD and the perception of PQoL of patients.

Table 6 and Appendix Fig. 4 show that variable X: SCHD (severe, moderate, or mild disease) has a statistically significant relationship with the variable M: NSI (B = –0.6774, SE = 0.1321, t = –5.1271, *p* < 0.001), supporting hypothesis 1. It can also be seen that NSI is negatively correlated with PQoL (B = –2.1608, SE = 0.8247, t = –2.6202, *p* < 0.01) (an increase in NSI corresponds to a decrease in PQoL), supporting the hypothesis 2.

**Table 6.** **Total, direct and indirect effects (Model 6)**.

Predictive mediator effect of the number of surgical interventions (NSI) on the perception of Psychological Domain in QoL (PQoL) of patients according to Severity of Congenital Heart Disease (SCHD)							
		Coeff	Se	T	*p*	LLCI	ULCI
Path a	SCHD (X)	–0.6774	0.1321	–5.1271^***^	0.0000	–0.9381	–0.4167
Path b	NSI (M)	–2.1608	0.8247	${\mathrm{–1.9947}}^{*}$	0.0095^**^	–3.7881	–0.5335
Path c	Total effect of X on Y	0.9565	1.4855	0.6439	0.5205	–1.9747	3.8877
Path c’	Direct effect of M on Y	–0.5073	1.5650	–0.3241	0.7462	–3.5955	2.5809
Indirect effect of X on Y ab 95% bootstrap confidence interval		Effect	BootSE				BootLLCI
NSI		1.4638	0.8178			0.2649	3.4237

SE, size of effect; LLCI, lower limit of confidence interval; ULCI, upper limit of confidence interval; QoL, quality of life; Boot, resampling simulation. The number of sample simulations for bias correction of confidence intervals: Level of confidence for all confidence intervals: 95. ^*^p < 0.05; ^**^p < 0.01; ^***^p < 0.001.

**Fig. 4.**
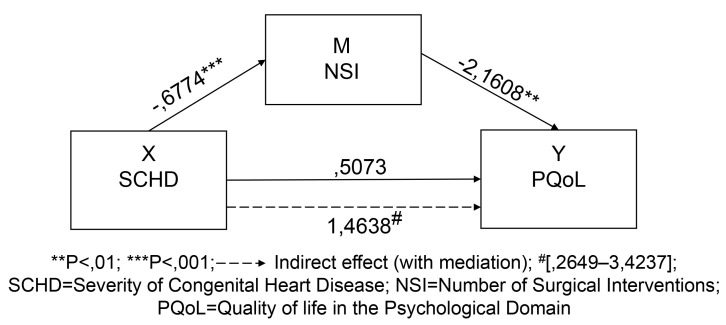
**Model 6: Unstandardized path coefficients for mediation**.

The direct effect between the variables X: SCHD and Y: PQoL shows a positive association (B = –0.5073, SE = 1.5650, t = –0.3241, *p* > 0.05), although this relationship is not statistically significant (the B value is negative because of the characteristics of variable X: discreet, but the meaning of the statistical relation is positive). As for the point estimate of the specific indirect effect through the mediating variable M (NSI) (X- > M- > Y), the estimated indirect effect = 1.4638 points towards a decrease in PQoL: the numerical values reveal an increase, but the statistical significance of this relation corresponds to a decrease due to the characteristics of the variable X: discreet, supporting the hypothesis 3. The bias-adjusted confidence interval of the product of this relation between variables of 95%, calculated according to the resampling simulation method between 2649 and 34,237, does not include zero, thus rejecting the null hypothesis and providing evidence of a significant mediator effect, as well as of the pertinence of Model 6 of mediation.

## 4. Discussion 

This study aimed to investigate the impact of selected clinical variables (namely, the number of surgeries) as mediators of the relationship between CHD (including severity and presence/absence of cyanosis) and patients’ perception of QoL. The available body of research on the perception of QoL in CHD patients is already extensive. Previous studies have highlighted that the increased survival of the CHD population due to advances in pediatric cardiac care has led to a rise in lifelong medical, psychosocial, and behavioural challenges, raising concerns about these patients’ well-being and perceived QoL.

However, new evidence is still needed, particularly regarding the mediation effects of contextual variables. More comprehensive and realistic explanatory models could benefit researchers, clinicians, and CHD patients. The prevalence of CHD is estimated at 0.3% in the global population of approximately 4.4 billion adults, which translates to approximately 13 million adult CHD survivors worldwide [37, 38]. Based on these figures, we can expect approximately 25,000 adults with CHD in Portugal. To the best of our knowledge, this is the first study to examine the importance of certain clinical variables as mediators of the impact of illness on QoL in CHD patients.

We tested 10 models to assess the predictive mediating effect of NSI on QoL in multiple domains (physical, psychological, social relationships, environmental, and general) in CHD patients while also considering the TCHD and SCHD criteria separately. Before proposing these models, we thoroughly reviewed the existing research on QoL and the impact of surgical interventions on these patients [4, 25, 39, 40, 41, 42]. Following a strategic plan, we also performed several statistical tests beforehand to identify potential models for further investigation. We then used the methodology of multiple bootstrap simulations proposed by Hayes [36] to more thoroughly examine these models.

As a primary finding from this analysis, we were able to confirm that the presence of cyanosis and the severity of CHD relate to the number of surgical interventions performed in patients. Secondly, we also confirmed that NSI negatively relates to patients’ quality of life in psychological and social relationship’s dimensions. Both facts could be expected and have been described previously in the literature. But the most relevant finding of this study is that the detrimental effect of cyanosis and severity of illness in QoL, in psychological and social relationship’s dimension, is fully explained by the mediating effect of the number of surgical interventions performed in the patients, in a pure mediation effect.

Our results from the tests of the mediation models suggest that patients, particularly those with more severe and cyanotic CHD, who undergo a greater number of surgical interventions, have an increased risk of perceiving negative QoL in the Psychological and Social Relationships domains. Interestingly, we did not find evidence of a mediation effect in other domains of QoL, such as Physical, Environmental, and Global. It appears that patients are more aware of the psychological and social consequences of the disease when evaluating their QoL, perhaps because they associate these consequences with a loss of freedom or control, which can indicate resilience, mental health, or some form of stress or exhaustion.

These findings provide a comprehensive foundation for planning and organizing effective interventions by healthcare professionals, including psychologists, for CHD patients. One of the strengths of this study is the substantial sample size of CHD patients, which is comparatively large compared to other studies in this area. Another positive aspect is the diverse range of variables analyzed and the extensive evaluation of sociodemographic factors, including age and the diversity of CHD diagnoses. Additionally, this study is advantageous because it allows for analyses of the relationship between patients’ perceptions of QoL and clinical and procedural variables.

A poorer perception of QoL has been reported by patients submitted to a greater number of surgeries in the Psychological and Social Relationships Domains [25, 39, 40]. The results of this study suggest that NSI can be considered a mediator variable, which explains the mechanism by which the two independent predictors under focus (and SCHD) influence the dependent variables (perceptions of QoL in various domains). Mediators explain how external physical events assume inner psychological significance [43]. In this study, NSI serves as a mediating variable, explaining how TCHD and SCHD impact certain domains of QoL, specifically the Psychological and Social Relationships domains.

NSI may explain how CHD impacts the Psychological domain of QoL through the feeling of threat to life and fragility associated with surgeries and the restrictions of freedom and autonomy [25, 39, 40]. NSI can also explain how CHD impacts social relationships since the recurrent hospitalizations and the associated circumstances can lead to restrictions on access to the family environment to full social support [25, 39, 41], factors that are strong predictors of QoL.

Our study has some limitations. On the one hand, the diversity of the analyzed variables may pose challenges in grouping all the results together. However, other variables could be introduced, such as the effect of the number of surgeries on body aesthetics, the ease or difficulty in physical exercise (sport), the social parameters regarding the difficulties that the severity of the disease may pose in obtaining a driving license, requirements for obtaining bank credits or health insurance, or difficulty in obtaining or maintaining employment due to potential absence from work as a result of new surgeries or treatments. Another variable that would be interesting to consider in the future is the age of patients at the last surgery, as it is reasonable to expect that the most recent memory of surgical interventions could influence the personal perception of QoL.

Given these findings and the need to plan and organize effective interventions by healthcare professionals, including psychologists, for CHD patients, it would be helpful to consider and include variables that may have a “dampening” effect on the impact of CHD on patients’ perception of QoL. This could include providing patients better access to family support, psychosocial support, integration, and academic performance. Raising awareness among healthcare professionals, education professionals, families, and the community could help ensure the inclusion of these variables and a greater capacity to involve family members and key members of the social network of patients in healthcare services.

## 5. Conclusions 

Childhood CHD is a complex and multifaceted condition that poses a significant challenge for patients, their families, and healthcare professionals. Here, we explored the impact of CHD on patients’ QoL and investigated the mediating variables that may influence this relationship. We found that patients with more severe and cyanotic diseases typically have more surgical interventions, which increases the risk of negative outcomes and harms patients’ perception of QoL. These findings will help health professionals and psychologists treating childhood CHD patients.

## Data Availability

The datasets used and/or analyzed during the current study are available from the corresponding author on reasonable request.

## Appendix

See Figs. 2,3,4.
